# A Tissue Engineering Acoustophoretic (TEA) Set-up for the Enhanced Osteogenic Differentiation of Murine Mesenchymal Stromal Cells (mMSCs)

**DOI:** 10.3390/ijms231911473

**Published:** 2022-09-29

**Authors:** Hui Zhang, Nirina Beilfuss, Urszula Zabarylo, Kay Raum, Regina Puts

**Affiliations:** 1 Center for Biomedicine, Charité-Universitätsmedizin, 12203 Berlin, Germany; 2 Berlin Institute of Health (BIH) Center for Regenerative Therapies, Charité-Universitätsmedizin, 13353 Berlin, Germany

**Keywords:** acoustophoresis, ultrasound, tissue engineering, patterned 3-D construct, mesenchymal stromal cells, osteogenic differentiation, bone regeneration

## Abstract

Quickly developing precision medicine and patient-oriented treatment strategies urgently require novel technological solutions. The randomly cell-populated scaffolds usually used for tissue engineering often fail to mimic the highly anisotropic characteristics of native tissue. In this work, an ultrasound standing-wave-based tissue engineering acoustophoretic (TEA) set-up was developed to organize murine mesenchymal stromal cells (mMSCs) in an in situ polymerizing 3-D fibrin hydrogel. The resultant constructs, consisting of 17 cell layers spaced at 300 µm, were obtained by continuous wave ultrasound applied at a 2.5 MHz frequency. The patterned mMSCs preserved the structured behavior within 10 days of culturing in osteogenic conditions. Cell viability was moderately increased 1 day after the patterning; it subdued and evened out, with the cells randomly encapsulated in hydrogels, within 21 days of culturing. Cells in the structured hydrogels exhibited enhanced expression of certain osteogenic markers, i.e., Runt-related transcription factor 2 (RUNX2), osterix (Osx) transcription factor, collagen-1 alpha1 (COL1A1), osteopontin (OPN), osteocalcin (OCN), and osteonectin (ON), as well as of certain cell-cycle-progression-associated genes, i.e., Cyclin D1, cysteine-rich angiogenic inducer 61 (CYR61), and anillin (ANLN), when cultured with osteogenic supplements and, for ANLN, also in the expansion media. Additionally, OPN expression was also augmented on day 5 in the patterned gels cultured without the osteoinductive media, suggesting the pro-osteogenic influence of the patterned cell organization. The TEA set-up proposes a novel method for non-invasively organizing cells in a 3-D environment, potentially enhancing the regenerative properties of the designed anisotropic constructs for bone healing.

## 1. Introduction

The approaches traditionally used in clinical top-down tissue engineering (TE), which employ three crucial components, i.e., scaffolds, cells, and biological factors [1], have achieved certain success in the regeneration of thin or avascular tissues, such as skin [2] and cartilage [3]. However, the restoration of organ complexity using the top-down approach, where cells randomly populate the designed tissue construct, along with poor perfusion and a lack of vascularization, often remains out of the question. The newly emerging bottom-up approaches attempt to mimic the micro-histoarchitecture of tissue, thereby preserving its unique functionality [4]. The resultant 3-D functional units can reconstruct the repaired organ as building blocks recreating the desired tissue pattern [5,6,7]. The 3-D microstructural histoarchitecture is defined through the extracellular matrix (ECM), a fibrous network deposited and continuously remodeled by cells in a persistent interplay [8]. The ECM properties, fiber composition, and fiber orientation are sensed by cells; these factors, in turn, determine the fate of the cells, e.g., cell cycle progression, differentiation, and migration. This is achieved via the actin–myosin core, which transmits cues on cellular organization over long ranges [9,10,11].

There are a number of methods that attempt to reconstruct organ structure in a bottom-up manner, with one of them being cell sheet technology (CST). CST allows recreating structures of multiple cell types, albeit with limited complexity and at the cost of intensive labor, with the requirement for direct manipulation of the cell-containing material [12,13]. Three-dimensional bioprinting is another promising technology for recreating tissue histoarchitecture. This method usually positions cells in a layer-by-layer manner; however, this approach often suffers from poor cell survival, irregular cell deposition within the construct, clotting of cells in the nozzle, time-consuming production, and high costs [14,15]. 

The application of external physical fields, i.e., magnetic [16,17], electrical [18], and acoustic forces [19,20,21], as novel regenerative medicine strategies for bone healing has been intensively researched by the scientific community. Another TE approach for the fabrication of regenerative constructs is the self-assembly of cells and biomolecules into a functional scaffold. Hydrogels generated from self-assembling ionic peptides were shown to recreate the specific nanostructure of an ECM, supporting cell adhesion and the proliferation of osteoprogenitors [22]. An organized tissue-specific 3-D structure can be achieved via the application of ultrasound, which enables the positioning of unlabeled cells within seconds with micrometer precision [23]. The propagated ultrasound waves result in a standing wave pattern upon encountering the reflective interface, which separates the particles present in the medium according to their acoustic properties, a phenomenon known as acoustophoresis. An in situ polymerizing hydrogel can be further used to immobilize the cells in a multilayered patterned 3-D construct. The distance between the layers and the shape of the construct can be varied at need by adjusting the acoustic frequency. This method has been previously used to create vascularized 3-D constructs [24,25,26], to mimic inter-layered brain-like conditions [27], and to demonstrate the enhanced contractility of aligned differentiated myocytes [28], among many other applications.

In this work, we describe a tissue engineering acoustophoretic (TEA) set-up for the organization of murine mesenchymal stromal cells (MSCs) in an in situ polymerizing fibrin hydrogel. MSCs are multipotent cells that, due to their potential for broad differentiation into chondrogenic, adipogenic, and osteogenic lineages, as well as their immunomodulatory properties, show great promise in regenerative therapies for both autologous and allogeneic transplantation [29]. In this work, the TEA set-up was characterized, and optimal acoustic parameters were determined. The multilayered patterned constructs maintained axis-dependent anisotropic cell organization within 10 days of culturing, and the enhanced osteogenic differentiation of the cells in the patterned constructs was observed.

## 2. Results

### 2.1. Speed of Sound (SOS) Measurement

In order to design the acoustophoretic chamber with the optimal height for the constructs, the SOS in non-polymerized fibrinogen was measured, and the impact of particles, i.e., red fluorescent beads (d = 12 µm) and cells suspended in fibrinogen at a concentration of 5 mg/mL, was analyzed (Table 1). The established SOS value of 1494 m/s [30] in water was chosen for a comparative control. The SOS values for the unpolymerized fibrinogen, as well as for fibrinogen with cells or beads, were slightly but significantly higher than that for water at 24 °C. 

For the TEA experimental work, an excitation frequency of 2.5 MHz, which is equal to the resonance frequency of the piezo-crystal and enables the highest ultrasound transmission into the acoustophoretic chamber, was chosen. Given the chosen frequency and using Equation (1), we expected to receive 17 layers spaced at 300 µm in the acoustic chamber, with a height of 5.1 mm.

(1) $$f=n*\frac{SOS}{\left(2*H\right)}$$

where n, f, SOS, and H are the number of nodes, frequency, speed of sound, and height of the acoustophoretic chamber, respectively.

### 2.2. Ultrasound Wave Mode and Transmission Voltage in the TEA Set-Up

**Figure 1:**
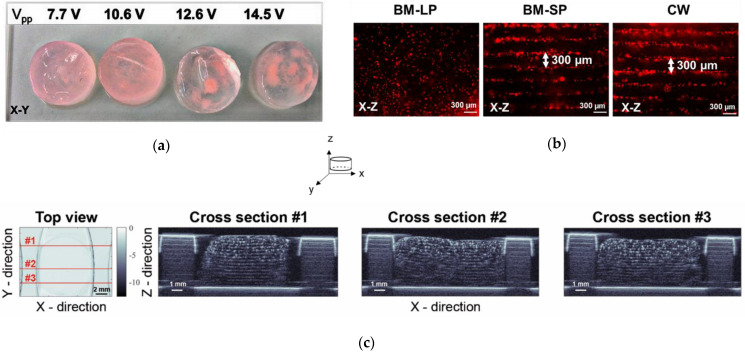
(**a**) Photographs of hydrogels from the top (X-Y) at increasing transmission peak-to-peak voltage (Vpp); (**b**) fluorescence images of red beads (12 µm) patterned at different ultrasound wave modes, where BM-LP, BM-SP and CW stand for ultrasound burst mode short pulses, burst mode long pulses and continuous wave, respectively; (**c**) SAM200Ex C-scan mode acoustic microscopy images of patterned fibrin gels at three different X-Z projections. The representative images are of the experiments repeated three times.

Red fluorescent beads were patterned at the frequency of 2.5 MHz and with transmission peak-to-peak voltage (Vpp) values of 7.7, 10.6, 12.6, and 14.5 V in fibrin gels. With increasing transmission voltage, the agglomeration of beads in the constructs was observed (Figure 1a), and the transmission voltage of 7.7 V was chosen as optimal for the following experiments. Next, the organization of beads was tested in different ultrasound modes: i. burst mode with long pulses of 280 µs, 35 cycles (BM-LP); ii. burst mode with short pulses of 20 µs, 35 cycles (BM-SP); and iii. continuous wave (CW). Imaging of fixed and cut-in-half hydrogels revealed that CW and BM-SP resulted in a visually similar organization of beads spaced at approximately 300 µm, whereas BM-LP led to randomly distributed particles (Figure 1b). The beads organized in hydrogels were then additionally visualized via scanning acoustic microscopy (SAM). C-scan images confirmed the homogenous patterning of beads throughout the entire gel (Figure 1c).

### 2.3. Cell Patterning in 3-D Fibrin Hydrogels

**Figure 2:**
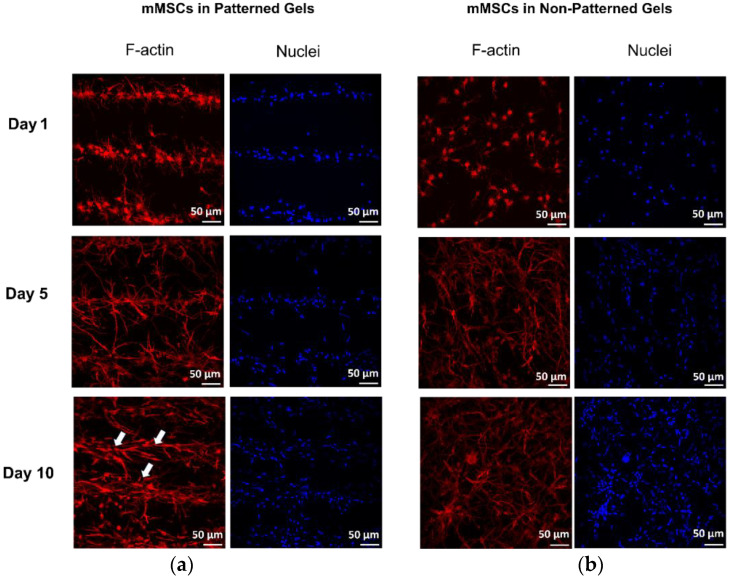
(**a**) mMSCs organized in fibrin hydrogels preserved a layered structure within 10 days of culturing in osteogenic degradation-free media with aprotinin (OMA); (**b**) filamentous actin (F-actin) bundles appear thicker and more intensive in the patterned gels (white arrows) in comparison to the thin and diffuse fibers in non-patterned gels.

The established acoustic parameters were used to pattern mMSCs in fibrin gels and, as was expected, the distances corresponded to approximately 300 µm, and bands were clearly formed (Figure 2a). 

Confocal imaging of cell organization in fibrin hydrogels revealed that cells preserved the layered structure within 10 days of culturing (Figure 2a). Filamentous actin (F-actin) fibers appeared thick in the patterned hydrogels, in comparison to thin and diffuse fibers in the non-patterned constructs (Figure 2b). Cell morphology appeared elongated in the non-patterned hydrogels, whereas the cells in the organized constructs exhibited a star-shaped morphology. In addition to the layered alignment of cells, the interlayer communication of cells was observed in the patterned gels.

### 2.4. Impact of Patterning on Cell Behavior

**Figure 3:**
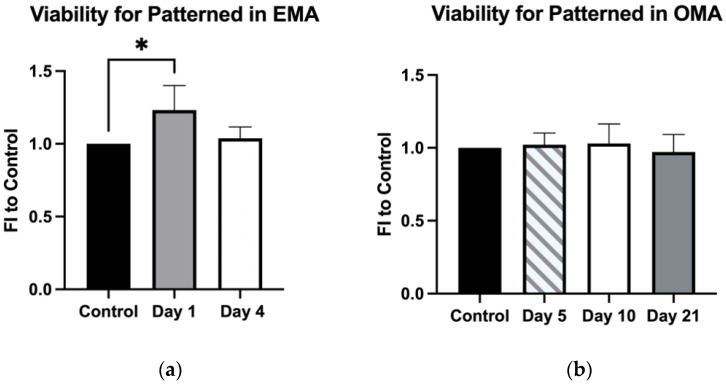
Cell viability of mMSCs measured by Presto Blue assay in patterned and non-patterned gels, cultured either in expansion degradation-free media with protinin (EMA) (**a**) or osteogenic degradation-free media with aprotinin (OMA) (**b**). * represents *p* ≤ 0.05. FI stands for fold induction compared to control (non-patterned gels).

Cellular viability was evaluated via Presto Blue assay, and a subtle but statistically significant increase in mMSC activity was observed the next day after the cell organization in hydrogels cultured in expansion degradation-free media with aprotinin (EMA) (Figure 3a). This effect subsided on the fourth day of culture. No effect on cell viability was observed in gels cultured long-term in osteogenic degradation-free media with aprotinin (OMA) (Figure 3b).

### 2.5. Gene Expression in the Patterned Fibrin Constructs in Media with and without Osteogenic Supplements

The mMSCs were cultured either in expansion degradation-free media with aprotinin (EMA) or in osteogenic degradation-free media with aprotinin (OMA) for 5 days and compared. These two types of media were chosen to investigate the outcomes of cell patterning itself on gene expression in an osteogenic-factor-independent manner. 

During the differentiation of MSCs into osteoblasts, osteogenic genes experience time-dependent expression [31,32]. Runt-related transcription factor 2 (RUNX2), an indispensable transcription factor for osteoblastic differentiation [33], is up-regulated early during the differentiation process, and its increased expression is maintained until at least day 21 in vitro. Osterix (Osx) functions downstream from RUNX2 and is another essential transcription factor for bone formation [34]; its expression starts increasing around day 7 of osteogenic differentiation. The expression of bone matrix protein osteopontin (OPN) shows a cyclic expression that increases around day 4, goes down on day 7, and then again peaks on day 14, persisting during the mineralization process. Osteocalcin (OCN) is a downstream target of RUNX2 [35] and an important hormone for the whole body metabolism [36]. OCN is a late-response gene whose expression starts taking off after day 7 of MSC differentiation and increases until at least day 21. Collagen-1 alpha1 (COL1A1) is a major protein component of bone and is an early marker of osteoprogenitors; it gradually increases throughout cell differentiation, reaching its maximum at three weeks of culturing. Osteonectin (ON), a critical player in the bone extracellular matrix assembly [37], is steadily elevated throughout osteogenesis. Alkaline phosphatase (ALP), an extracellular protein regulating bone mineralization [38], gradually increases until day 7; its expression starts dropping during the beginning of the mineralization phase, reaching its minimum at day 21. 

**Figure 4:**
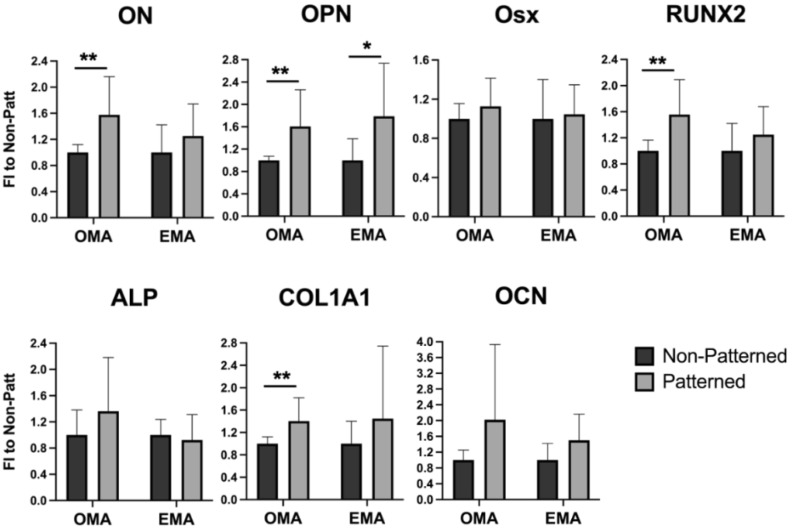
Expression of osteogenic genes in mMCSs organized in patterned 3-D fibrin hydrogels, cultured in either expansion degradation-free media with aprotinin (EMA),or osteogenic degradation-free media with aprotinin (OMA), quantified by qRT-PCR on day 5. Gels with randomly encapsulated cells (non-patterned) served as a negative control. * and ** represent *p* ≤ 0.05 and *p* ≤ 0.01, respectively.

The enhanced expression of osteogenic markers, i.e., ON, OPN, COL1A1, and RUNX2 (Figure 4), in the patterned constructs cultured in the osteoinductive media (OMA) was observed on day 5. Interestingly, OPN gene expression was also up-regulated in patterned gels cultured in non-osteogenic conditions (EMA), suggesting that the patterning alone has a pro-osteogenic influence. Two-way ANOVA analysis also revealed an effect of cells organized in a patterned manner on the expression of the OCN gene (*p* = 0.0460). However, the pairwise comparison did not confirm any statistically significant changes. 

**Figure 5:**
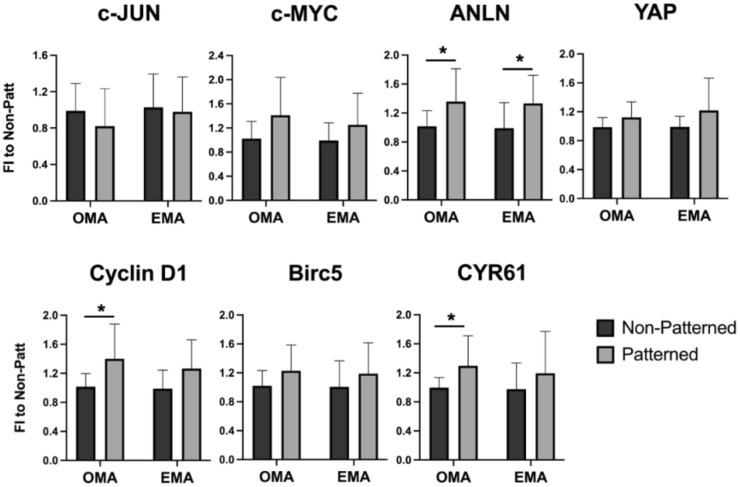
Expression of cell cycle progression genes in mMCSs organized in patterned 3-D fibrin hydrogels, cultured in either expansion degradation-free media with aprotinin (EMA), or osteogenic degradation-free media with aprotinin (OMA), quantified by qRT-PCR on day 5. Gels with randomly encapsulated cells (non-patterned) served as a negative control. * represents *p* ≤ 0.05.

Genes influencing cell cycle progression, i.e., Cyclin D1, anillin (ANLN), and cysteine-rich angiogenic inducer 61 (CYR61), were also affected by patterning (Figure 5) in the osteogenic media OMA, and ANLN was also enhanced in the expansion media EMA. Two-way ANOVA analysis showed that the cell patterning had a significant effect on the expression of MYC proto-oncogene protein (c-MYC) and yes-associated protein (YAP) genes, *p* = 0.0408 and *p* = 0.0494, respectively. However, the pairwise comparison did not result in statistical significance. The expression of early-response transcription factor JUN (c-JUN) and baculoviral IAP repeat containing 5 (Birc5) survival genes was not influenced by the cell organization. 

### 2.6. Expression of Osteogenic Genes in the Patterned Fibrin Constructs Cultured for 10 Days in Osteoinductive Media

**Figure 6:**
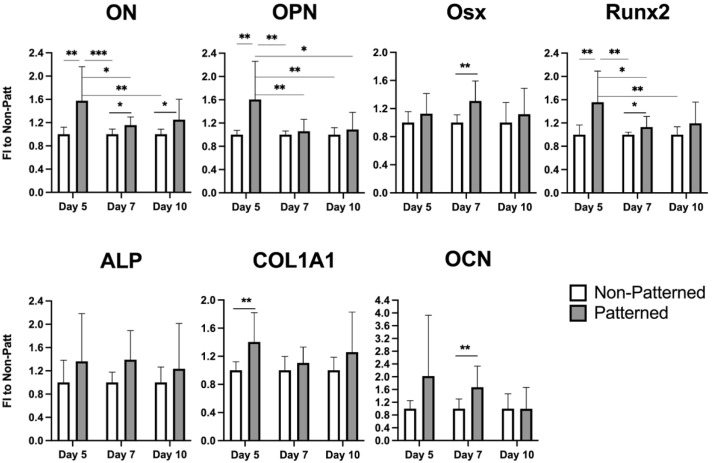
Expression of osteogenic genes on days 5, 7, and 10 in patterned hydrogels cultured in osteogenic degradation-free media with aprotinin (OMA). Gels with randomly encapsulated cells (non-patterned) served as a negative control. *, **, and *** represent *p* ≤ 0.05, *p* ≤ 0.01, and *p* ≤ 0.001, respectively.

The degradation-free osteogenic media (OMA) was then selected for long-term culturing of the patterned constructs at additional time points. Days 7 and 10 were chosen to monitor the progression. The most pronounced effects for all genes showing elevated expression in patterned hydrogels were observed on day 5 (Figure 6). Osx, RUNX2, and OCN were also slightly but significantly enhanced on day 7, and only the ON gene was augmented at every time point. Two-way ANOVA analysis showed a statistically significant effect (*p* = 0.0452) of patterning on the gene expression of ALP; however, this was not observed for pairwise comparison. 

## 3. Discussion

Sorting cells in acoustophoretic fields is a promising method to rapidly and non-invasively organize cells at the required positions. The application of a tissue engineering acoustophoretic (TEA) set-up for the patterning of murine mesenchymal stromal cells (mMSCs) was developed in the current study to enhance the osteogenic properties of 3-D fibrin constructs for bone regeneration.

The ultrasound-based TEA set-up organized mMSCs within seconds in parallel bands in an in situ polymerizing fibrin gel. The optimized acoustic parameters for the set-up were a frequency of 2.5 MHz in continuous wave mode, with a 7.7 V peak-to-peak transmission voltage. As a result of the measured SOS in fibrinogen and the selected acoustic parameters, 17 cell layers spaced at approximately 300 µm were formed. The acoustic imaging of the 3-D constructs showed a sufficiently homogenous distribution of the fluorescent beads throughout the entire scaffold (Figure 1c). The 10-day cultivation of the constructs revealed preserved patterned cell organization in the parallel layers (Figure 2a), with the F-actin bundles in a preferred orientation parallel to the cell bands. 

The application of ultrasound at low intensities has been shown to have a pro-regenerative influence on some soft tissues [39] and bone [40]. However, an increase in the intensity levels has a cytotoxic effect, which has promise for cancer therapies [41,42]. The effect of the TEA set-up on cell viability was also evaluated in this study. In the patterned constructs, enhanced cell viability was observed 24 hours after the acoustophoresis (Figure 3a). This observation might have been due to the higher probability of cell interactions (Figure 2a, Day1), in comparison to the singled cells in the randomly encapsulated hydrogels (Figure 2b, Day1). The prominence of cell–cell contacts for proliferation has been previously discovered to be regulated via the PI3K signaling pathway [43]. The effect was no longer detectible 4 days after the culturing in expansion media and was not observed during the long-term cultivation in osteogenic media; most likely, the effect evened out due to the increasing contact inhibition of growth [44,45] and the gas/nutrient gradients further discussed in detail below. However, the expression of Cyclin D1 and CYR61 in OMA, and ANLN in both EMA and OMA, was elevated on day 5 in the patterned gels (Figure 5). Cyclin D1, CYR61, and ANLN proteins are known to be critical for cell proliferation via the regulation of cell cycle progression [46,47] and cytokinesis [48].

Gene expression analysis revealed that certain osteogenic markers, i.e., RUNX2, OCN, OPN, Osx, ON, and COL1A1, were enhanced on at least one of the selected days (Figure 6). The effects were the most pronounced on day 5 and were gradually reduced with the cell culturing on days 7 and 10. The intensity of the effect could have been alleviated due to inevitable compromised cell-survival conditions in the core of the 3-D construct. This could be improved in future experimental work by positioning the constructs in the perfusion bioreactor to minimize gradients of gas and nutrients [49,50,51]. Alternatively, the co-application of low-intensity pulsed ultrasound (LIPUS), previously used to enhance cell survival both in 2-D [52,53,54] and 3-D environments [55,56,57,58], could be attempted. An ultrasound-based portable bioreactor achieving both cell-structured scaffolds and pro-regenerative stimulation is currently being developed in our laboratory. Only the expression of the ON or SPARC gene was enhanced on all three days tested (Figure 6). The ON protein influences bone formation via the regulation of procollagen processing and collagen cross-linking [37].

Interestingly, mMSCs cultured in normal expansion media without the addition of pro-osteogenic supplements also experienced a higher expression of the OPN gene on day 5 (Figure 4), in comparison to the cells freely populating hydrogels, implying that the patterning alone promoted the enhanced expression of the osteogenic marker. Cellular surroundings, i.e., architecture and mechanical properties, dictate cell attachment, cytoskeleton organization, and shape; these surroundings play crucial roles in stem cell commitment [59,60,61,62]. Several attempts were undertaken to direct the differentiation of MSCs via tuning the physical properties of the cellular environment. As an example, aligned carbon-nanotube-derived topographical changes, enforcing an elongated cell morphology of adipose-derived MSCs, promoted the expression of certain myogenic markers, i.e., PAX7 and MYF5 [63], and suppressed the expression of certain osteogenic markers, i.e., OPN, Runx2, ALP, and COL1A1, when compared to the randomly oriented substrates. In contrast, human MSCs grown on aligned carbon nanotubes that constrained the cells to stretched and elongated shapes were found to have an osteoinductive influence [64]. Both of the studies used media without any lineage-directing supplements, and it is not clear what the difference in observations can be attributed to. It was determined by the authors of [63] that the expression of MyoD, which is the key transcription factor for myogenic commitment [65], was not affected by the cell alignment, suggesting that myogenic differentiation cannot be directed efficiently via matrix organization. In our work, cells seeded randomly in the hydrogels acquired more elongated morphology, with thin F-actin fibers (Figure 2b, Days 5 and 10), whereas mMSCs in the patterned scaffolds had a star-shaped morphology with pronounced stress filaments (Figure 2a, Days 5 and 10); in addition, a more enhanced expression of osteogenic markers was observed. These findings are in line with a study showing that an MSC cell constricted to a star-shaped region had a pronounced osteogenic phenotype in comparison to the MSCs seeded on flower-shaped forms, which were directed into an adipogenic lineage [66]. The star-shaped cells formed intense stress fibers, stretching from the pointed regions, and experienced high actomyosin contractility. In the study by Graziano et al. in 2007 [67], a scaffold morphology with a large number of microcavities enhanced the osteogenic differentiation of pulp-derived MSCs via an increase in the adhesion area. An increasing cell area and F-actin fiber thickening were previously shown to be associated with enhanced osteogenic differentiation [68]; however, the cell area increase was similarly observed for cells undergoing adipogenic differentiation. These results imply that not only the cell area but also the local shape cues of the cell are determinant factors of cell fate commitment. 

Due to poor long-term cell survival in a 3-D environment, this study was limited to a 10-day culturing period. On day 15, a very low viable cell number was observed in the middle of the construct. As mentioned above, this could be overcome via the use of additional stimulatory techniques, such as ultrasound, or by culturing the constructs in perfused bioreactors. Additionally, the osteogenic differentiation was only evaluated via the gene expression of osteogenic markers, without an assessment of the properties of the formed ECM. This is planned in future experimental work, where the effect of patterning on the deposition of ECM proteins and matrix mineralization, as well as gel anisotropy, will be evaluated. The changes in the mechanical properties of the designed constructs are also planned to be tested. In this work, only relative changes in gene expression were quantified, where each data duplicate was normalized to the mean value of the non-patterned control. Since the time points were not chosen with large enough intervals, no drastic changes were observed in gene expression between the selected days. This hindered the use of absolute values for the data analysis to demonstrate the effect of cell organization and could be another limitation of this study. Most certainly, the TEA set-up described here cannot be directly used for the creation of 3-D bone regenerative constructs, due to the complex hierarchy of bone structure. However, this set-up can be combined in the future with other high-precision techniques, e.g., 3-D bioprinting [69,70,71], to grant regenerative constructs the desired tissue intricacy. In closing, the TEA set-up has the potential to be adapted for in situ applications during surgical procedures, where gel components containing cells and/or biofactors can be injected directly into a deteriorated organ site and organized via acoustic fields in the required pattern. Injectable smart materials with tunable mechanical properties [72,73] and non-invasive technologies, such as TEA, are of critical importance for this indispensable advancement in personalized medicine.

## 4. Materials and Methods

### 4.1. Tissue Engineering Acoustophoretic (TEA) Set-Up

**Figure 7:**
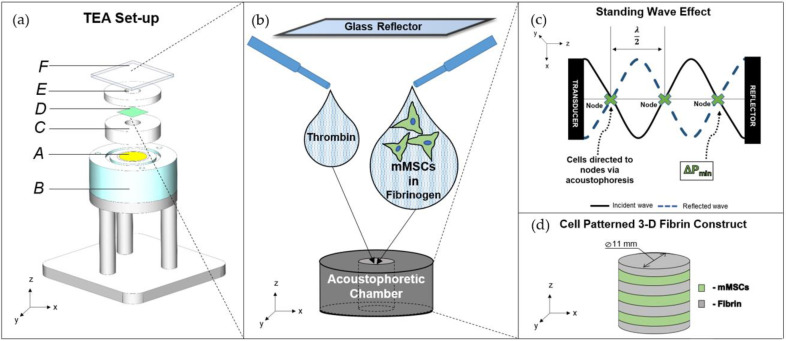
Tissue engineering acoustophoretic (TEA) set-up: (**a**) SOLIDWORKS software-designed drawing of the TEA set-up, displaying the key components: piezoelectric ceramic disk (A), 3-D-printed stand (B), coupling chamber (C), sealing film (D); acoustophoretic chamber (E) and glass reflector (F); (**b**) zoom-in into the acoustophoretic chamber; (**c**) principle of standing wave effect and acoustophoresis-directed cell patterning; (**d**) three-dimensional patterned fibrin cell construct.

The TEA set-up (Figure 7a) consisted of: a planar piezoelectric ceramic disk (A) with a diameter of 20 mm, a thickness of 0.8 mm, and a resonance frequency of 2.5 MHz (STEMiNC, Davenport, FL, USA) embedded into a 3-D-printed plexiglass-stand (B); a 10 mm tall Teflon block (C), i.e., a coupling chamber, placed on the ultrasound probe and filled with a coupling gel; a 5 mm tall Teflon block, i.e., an acoustophoretic chamber (D), placed on top of the coupling chamber and sealed with a sterile thin polystyrene film (E) (Excel Scientific Inc., Victorville, CA, USA); a 2 mm glass reflector (F), positioned on top of the acoustophoretic chamber, ensuring the generation of a standing wave pattern. Both Teflon blocks had central cylindrical compartments with a diameter of 11 mm, through which the blocks aligned. The hardware components included a function generator (Agilent Technologies Inc., Santa Clara, CA, USA) connected via a 15 dB attenuator to an amplifier (325LA, Electronics and Innovation, Rochester, NY, USA), which was connected to the ultrasound transducer and then supplied the desired acoustic pressure onto the TEA set-up. 

Fibrin hydrogel components and beads/cells were added into the acoustophoretic chamber (Figure 7b), giving them a slight swirl, while the acoustic signal was kept on. The reflector was immediately placed on top of the chamber. 

As a result of acoustophoresis and a reflector placed in the path of the propagating longitudinal ultrasound wave, a standing wave pattern was formed (Figure 7c), which consisted of alternating regions of pressure nodes and antinodes [24]. Particles with a positive acoustic factor, i.e., cells, were dragged to the nodes or low-pressure regions of the standing wave pattern, which can be described by Equation (1) (see the Section 2). Within 10 s, a polymerized and patterned fibrin construct was formed (Figure 7d). The signal was kept on for 10 min to let hydrogels polymerize fully, and the gels were moved aside to solidify for an additional 50 min at room temperature.

### 4.2. Fibrin Hydrogel Preparation 

Human fibrinogen (Sigma-Aldrich, St. Louis, MO, USA) was dissolved in phosphate-buffered saline (PBS) (Mediatech Inc., Manassas, VA, USA) without the addition of antibiotics, then warmed up for 3 min in a water-bath at 37 °C and filtered through a 0.2 µm PVDF filter (Carl Roth GmbH & Co. KG, Karlsruhe, Germany). The fibrinogen solution was stored in the incubator at 37 °C before resuspending cells or beads in it. Thrombin from bovine plasma (Sigma-Aldrich, St. Louis, MO, USA) was dissolved in PBS containing 5 mM CaCl_2_ (Mediatech Inc., Manassas, VA, USA) and stored on ice. Both gel components were equilibrated to room temperature right before adding them to the TEA set-up. The PBS used for the preparation of fibrin hydrogels did not contain antibiotics. Hydrogels were mixed in the acoustophoretic chamber: a total volume of 600 µL was received via the addition of 150 µL of thrombin to 450 µL of fibrinogen, with final concentrations of components in the gel of 1 U/mL and 5 mg/mL, respectively. To test the efficiency of acoustophoresis, red fluorescent polystyrene beads (Distrilab Particle Technology, Leusden, The Netherlands) with a diameter of 12 µm were used. For regenerative constructs, the cells were passaged as described above, centrifuged at 500 rpm for 5 min, and resuspended in fibrinogen. To ensure full polymerization, the constructs were left for an additional 50 min in the safety hood.

### 4.3. Measurement of Transmission Voltage 

**Figure 8:**
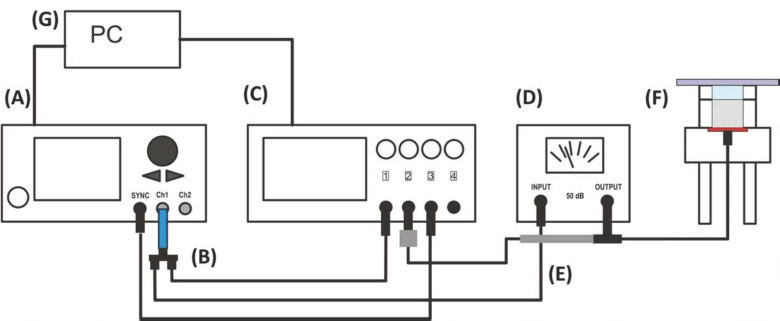
Schematic representation of the transmission voltage measurements in the TEA set-up: function generator (**A**), 15dB attenuator (**B**), oscilloscope (**C**), amplifier (**D**), oscilloscope probe (**E**), TEA set-up (**F**), and computer with software (**G**).

The acoustic pressure required for the organization of the cells was controlled via the transmission voltage at the ultrasonic transducer. For this purpose, the voltage signal was recorded on the oscilloscope via an oscilloscope probe connected to the T-piece of the amplifier output (Figure 8). All hardware components, settings, and Vpp values were controlled and recorded via a custom-made graphical user interface, developed with MATLAB software release 2020a (The MathWorks, Natick, MA, USA).

### 4.4. Speed of Sound Measurement

Determination of the speed of sound (SOS) in fibrin hydrogels was performed in the custom-made 3-D-printed chamber. This set-up consisted of an unfocused 5 MHz transducer emitting and detecting soundwaves, which was inserted at the bottom of a Teflon chamber containing the ultrasound transmitting media, i.e., fibrinogen at a concentration of 5 mg/mL, mimicking the initial point of the acoustophoresis. The upper part of the set-up was placed directly on top of the transducer to ensure the alignment of the cylindrical compartment with the transducer, building a cavity (H = 20.5 mm, d = 11 mm) for the sample medium. A steel plate covered the surface of the cavity to induce multiple reflections. SOS measurements were performed at room temperature. Double distilled water was used as a control, and values were compared to SOS ≈ 1494 m/s at room temperature (24 °C) [30].

The transducer was connected to a US-key single-channel ultrasound device (Lecoeur Electronique, Chelles, France) used for the transducer excitation and digitization of the received signal. Three echoes and reflections were generated, and the time difference between the second and the third one was analyzed through cepstral analysis [74]. Finally, the two-way travel distance, i.e., 2 times the height of the chamber (20.5 mm), was divided by the time delay obtained from the cepstral peak to calculate the SOS in the sample medium.

### 4.5. Cell Culture

Murine bone-marrow-derived mesenchymal stromal cell-line D1 (ATCC, CRL-12424TM) was cultured in expansion media composed of high-glucose Dulbecco-modified minimal essential medium (DMEM) (Life Technologies Limited Inc., Paisley, UK), 10% superior fetal bovine serum (FBS) (Sigma-Aldrich, St. Louis, MO, USA), 1% penicillin and 1% streptomycin (Pen-Strep) (Mediatech Inc., Manassas, VA, USA), 2 mM Glutamax (Life Technologies, Grand Island, NY, USA) in standard cultivation conditions (37 °C, 5% CO_2_, 95% humidity). On the day of producing the 3-D constructs, the cells were harvested at 70–80% confluency. For this purpose, PBS containing Pen-Strep and trypsin (both from PAN-Biotech GmbH, Aidenbach, Germany) were used to wash and detach the cells, respectively. Before the cells were encapsulated in fibrin gels, they were passed through a 40-µm cell strainer (Life Science Inc., Durham, NC, USA) and then counted to ensure no cell aggregation.

### 4.6. Construct Culturing in Expansion and Osteogenic Media 

After the gels were fully polymerized, they were taken out of the acoustophoretic chamber and washed 3 times with warm PBS. The fibrin constructs were then placed in 700 µL of expansion degradation-free media with aprotinin (EMA), which consisted of low-glucose DMEM media with sodium pyruvate (PAN-Biotech GmbH, Aidenbach, Germany), 10% FBS, 2 mM Glutamax, Pen-Strep, 5 mM CaCl_2_, and 50 µg/mL of aprotinin (Sigma-Aldrich, St. Louis, MO, USA). Osteogenic degradation-free media with aprotinin (OMA) had the same content as EMA and was additionally supplemented with osteogenic factors, i.e., 100 mM L-ascorbic acid (Sigma-Aldrich, St. Louis, MO, USA), 50 mM β-glycerophosphate (Sigma-Aldrich, St. Louis, MO, USA), and 5 nM dexamethasone (Sigma-Aldrich, St. Louis, MO, USA). The media were refreshed every two days. For all experiments, mMSCs were used at a concentration of 450 cells/µL.

### 4.7. Cell Viability Assay 

The Presto-Blue reagent (Invitrogen, Eugene, OR, USA) was mixed 1:10 *v*/*v* with the expansion media and added to the fibrin gels. The gels containing the reagent were incubated for 4 h at 37 °C, shaking at 150 rpm (DTS-2, Neolab Migge GmbH, Heidelberg, Germany). The absorbance was measured in duplicate at 570 nm with a reference wavelength of 600 nm on Tecan (Tecan Austria Inc., Groedig, Austria).

### 4.8. Fluorescent Staining

The constructs were washed in PBS and fixed for 20 min in a 4% paraformaldehyde (PFA) solution (ThermoFischer Scientific, Rockford, IL, USA). The gels were cut into 3 vertical slices similar in size to observe the cell layers. The cells were then permeabilized with 0.1% Triton X-100 (Sigma-Aldrich, St. Louis, MO, USA) for 10 min, followed by two rounds of washes in PBS. Fibrous actin was stained for 2 h by phalloidin-tetramethylrhodamine B isothiocyanate (Merck KGaA, Darmstadt, Germany) prepared in PBS containing 1% bovine serum albumin (BSA from Carl Roth GmbH & Co. KG, Karlsruhe, Germany) at a 1:40 ratio. The gels were then again washed 3 times in PBS. Nuclei were counterstained with Hoechst 33,542 (Invitrogen, Eugene, OR, USA) at 1:200 in PBS with 1% BSA for 2 h. The gels were finally washed 3 times in PBS and imaged either on a Leica TCS SP5 confocal microscope (Leica Microsystems, Wetzlar, Germany) or on an inverted AxioObserver (Carl Zeiss MicroImaging Co., Gottingen, Germany) fluorescent microscope. All of the incubation steps were performed at room temperature at 150 RPMs on the microplate shaker (DTS-2, NeoLab Migge GmbH, Heidelberg, Germany). Images were processed and analyzed using Image-J software version 1.53s (National Institute of Health, Bethesda, MD, USA).

### 4.9. Scanning Acoustic Microscopy (SAM)

The custom-built SAM-200Ex set-up [75] was used to analyze cell distribution throughout the entire construct. The gels were first fixed as described above and positioned into a custom-made metal stand, which was then placed into a tank containing degassed PBS. A C-scan was performed using a focused lithium niobate 40-MHz (NIH Resource Center for Ultrasound Transducer Technology, Los Angeles, CA, USA) transducer (f-number of 2.66, diameter of 3 mm). The images were reconstructed using custom-made MATLAB software release 2018b (The MathWorks, Natick, MA, USA) code.

### 4.10. Quantitative Real-Time Polymerase Chain Reaction Assay (qRT-PCR)

The fibrin constructs were lysed on the selected day, and messenger RNA (mRNA) was isolated using the NucleoSpin RNA II Kit (Machery Nagel, Düren, Germany) with some modifications. To ensure efficient cell lysis, fibrin gels were disintegrated by a sterile type 11 Cutfix^®^ scalpel (Carl Roth GmbH & Co. KG, Karlsruhe, Germany) and submerged into the lysis buffer; they were kept at −20 °C until frozen. Then, the gels were thawed and pipetted up and down rigorously. The isolated mRNA was transcribed to complementary DNA (cDNA) using qScriptTM cDNA SuperMix (Quanta Biosciences, Gaithersburg, MD, USA) on Mastercycler EP Gradient S (Eppendorf, Hamburg, Germany). The gene expression was measured using SYBRTM Green PCR Master Mix (Applied Biosystems GmbH, Darmstadt, Germany) on LightCycler 480 II (Roche Diagnostics GmbH, Mannheim, Germany). The primers were designed using Primer3 Input software version 0.4.0 (Whitehead Institute for Biomedical Research, Cambridge, MA, USA) and produced by TIB Molbiol (Berlin, Germany). The expression of all target genes was normalized to the expression of a housekeeping gene, glyceraldehyde 3-phosphate dehydrogenase (GAPDH). The primer sequences are summarized in Table 2.

### 4.11. Statistical Analysis

All measurements of TEA set-up characteristics were conducted in triplicate. The biological read-outs were repeated at least three times, with each trial having at least two replicas. All data were first tested for normality using the Kolmogorov–Smirnov test, which was then followed by a t-test or a Mann–Whitney test for pairwise comparison. For comparison of more than 2 groups, one-way ANOVA followed by a Tukey multiple comparison test was performed. For two-variable analyses, a two-way ANOVA and Šidák’s multiple comparison test were used. The significance level was set at *p* ≤ 0.5. Statistical analysis was performed with GraphPad Prism software version 9.1.1 (San Diego, CA, USA).

## 5. Conclusions

In this work, a tissue engineering acoustophoretic (TEA) set-up was developed and characterized to create 3-D anisotropic constructs with enhanced osteogenic properties for bone regeneration. The TEA set-up permits the production of 3-D building blocks for bone tissue engineering, with a high precision of cell positioning and reproducibility. The enhanced expression of osteogenic markers was observed in the TEA-patterned constructs cultured with osteogenic supplements. In addition, the expression of osteopontin (OPN) was elevated in the patterned gels even without the addition of the osteoinductive media. This quick and non-invasive method can be combined with other TE techniques, i.e., 3-D bio-printing, to create more sophisticated ex vivo constructs. Tissue complexity could also be achieved in the TEA set-up itself via changing: (i) the form of the construct, which is dependent on the selected shape in the Teflon chamber; (ii) the number of layers and the spacing length, which are dependent on the ultrasound frequency; (iii) the in situ polymerizing gel, which is dependent on the material properties; and (iv) the organization pattern, i.e., parallel planes, columns, and agglomerates, which is dependent on the number of transducers used. Methods permitting the long-term culturing of TEA-produced constructs, i.e., ultrasound-based stimulation and perfusion, are also in need of being further developed.

## Figures and Tables

**Table 1:** Speed of sound values measured by US-key device at 24 °C.

	H_2_O (dd)	Fibrinogen	Fibrinogen with Cells	Fibrinogen with Beads
**Mean, m/s**	1489.9	1498.3 ***	1497.9 ***	1501.0 ***
**SD**	0.8	0.5	0.7	1.7

H_2_O (dd) stands for double-distilled water. *** represents *p* values less than 0.0001 for three independent fibrinogen preparations.

**Table 2:** Primer sequences.

Gene	Forward	Reverse
**Cyclin D1**	5′–CGTGGCCTCTAAGATGAAGG–3′	5′–CCACTTGAGCTTGTTCACCA–3′
**BIRC5**	5′–CATCGCCACCTTCAAGAACT–3′	5′–AAAACACTGGGCCAAATCAG–3′
**CYR61**	5′–TGCTGTAAGGTCTGCGCTAA–3′	5′–AGGGTCTGCCTTCTGACTGA–3′
**YAP**	5′–AAGGAGAGACTGCGGTTGAA–3′	5′–CCTGAGACATCCCAGGAGAA–3′
**ANLN**	5′–TCAATAGCAGCAGTGTTCAGC–3′	5′–GATTTTGTGCCTCACGGTTT–3′
**c-MYC**	5′–GCTGTTTGAAGGCTGGATTT–3′	5′–CTCTGCTGTTGCTGGTGATAG–3′
**c-JUN**	5′–TGTTTGTTTGTTTGGGTGTCC–3′	5′–GAGGTTGGGGGCTACTTTTC–3′
**OPN**	5′–GCTTGGCTTATGGACTGAGG–3′	5′–GGGATGACATCGAGGGACT–3′
**OCN**	5′–CGCTCTGTCTCTCTGACCTC–3′	5′–GACTGAGGCTCCAAGGTAGC–3′
**ON**	5′–ACATTGCACCACACGTTTC–3′	5′–GGGACACATCAGAGGGAGAG–3′
**Osx**	5′–CCCTTCTCAAGCACCAATGG–3′	5′–AGGGTGGGTAGTCATTTGCATAG–3′
**RUNX2**	5′–TAAGAAGAGCCAGGCAGGTG–3′	5′–TAGTGCATTCGTGGGTTGG–3′
**ALP**	5′–AACCCAGACACAAGCATTCC–3′	5′–GAGAGCGAAGGGTCAGTCAG–3′
**COL1A1**	5′–GCCTCCCAGAACATCACCTA–3′	5′–GACTGTCTTGCCCCAAGTTC–3′
**GAPDH**	5′–TGCACCACCAACTGCTTAG–3′	5′–GAGGCAGGGATGATGTTC–3′

## Data Availability

The data presented in this study are available on request from the corresponding author.
